# The evolutionary landscape and expression pattern of plant lincRNAs

**DOI:** 10.1080/15476286.2022.2144609

**Published:** 2022-11-16

**Authors:** Li Chen, Qian-Hao Zhu

**Affiliations:** a School of Life Sciences, Westlake University, Hangzhou, China; b Institute for Biology, Plant Cell and Molecular Biology, Humboldt-Universität Zu Berlin, Berlin, Germany; c CSIRO Agriculture and Food, Canberra, Australia

**Keywords:** lincRNAs, evolution, plants, Arabidopsis, rice, transcriptional regulatory, TFs, development

## Abstract

Long intergenic non-coding RNAs (lincRNAs) are important regulators of cellular processes, including development and stress response. Many lincRNAs have been bioinformatically identified in plants, but their evolutionary dynamics and expression characteristics are still elusive. Here, we systematically identified thousands of lincRNAs in 26 plant species, including 6 non-flowering plants, investigated the conservation of the identified lincRNAs in different levels of plant lineages based on sequence and/or synteny homology and explored characteristics of the conserved lincRNAs during plant evolution and their co-expression relationship with protein-coding genes (PCGs). In addition to confirmation of the features well documented in literature for lincRNAs, such as species-specific, fewer exons, tissue-specific expression patterns and less abundantly expressed, we revealed that histone modification signals and/or binding sites of transcription factors were enriched in the conserved lincRNAs, implying their biological functionalities, as demonstrated by identifying conserved lincRNAs related to flower development in both the Brassicaceae and grass families and ancient lincRNAs potentially functioning in meristem development of non-flowering plants. Compared to PCGs, lincRNAs are more likely to be associated with transposable elements (TEs), but with different characteristics in different evolutionary lineages, for instance, the types of TEs and the variable level of association in lincRNAs with different conservativeness. Together, these results provide a comprehensive view on the evolutionary landscape of plant lincRNAs and shed new insights on the conservation and functionality of plant lincRNAs.

## Introduction

With the advancement of high-throughput sequencing technology, large parts of plant genomes are found to be transcribed, some encoding proteins but the majority being non-coding[1]. Among the non-coding transcripts, long non-coding RNAs (lncRNAs) are pivotal components and display spatiotemporal expression patterns and low expression levels compared to protein-coding genes (PCGs) [2,3]. LncRNAs have been shown to be important regulators of gene expression in plants and participate in a wide range of biological processes, including development and stress responses. For example, *HIDDEN TREASURE 1* (*HID1*) promotes photomorphogenesis under the continuous red light conditions and represses the expression of *PIF3* (*PHYTOCHROME INTERACTING FACTOR3*) by forming RNA-protein complex associated with the chromatin structure of *PIF3* promoter [4]. Although a small portion of lncRNAs have been functionally dissected, the functionality of most lncRNAs is still unknown.

Evolutionary studies of lncRNAs by comparative genomics could help us understand how sequence divergence contributed to lncRNA evolution, facilitate association of the primary sequence of lncRNAs with their functions and pinpoint critical functional regions of lncRNAs. Several studies on identified lncRNAs in animal genomes have revealed lower conservation and rapid evolution of lncRNAs in terms of primary sequences and gene structures and found short conserved regions within lncRNAs with a possible role in interacting with their partners, e.g. RBPs [5–7]. Similarly, comparison of lncRNAs identified by genome-wide surveys in maize, *Arabidopsis*, rice, and sorghum also revealed lower conservation of lncRNAs than that of PCGs [8]. Despite lower sequence conservation, the functionality of homologous lncRNAs in diverse plants is conserved as demonstrated by several examples, such as *HID1* [4], *IPS1* [9], and *ENOD40* [10,11]. In some cases, homologous sequences of lncRNAs found in a plant species could be identified at the conserved regions of another plant species, but that does not necessarily guarantee the potential homologous lncRNAs are expressed [5]. Comparison of lncRNAs in five monocots and five dicots revealed a higher sequence conservation at the level of intraspecies than that of interspecies [12,13]. Genome-wide identification of lncRNAs in rice and maize suggested a potential association between the conserved lincRNAs and agriculturally important traits [14]. LncRNAs with biased expression in different *Arabidopsis* ecotypes have functions in root development during phosphate starvation [15]. Moreover, compared with other transcripts, lncRNAs showed diverse patterns of molecular features (such as lower GC content) in 11 plant genomes [16]. Despite these observations, the evolutionary dynamics of lncRNAs in plants, including non-flowering plants, remain elusive and the expression pattern of homologous lncRNAs in different plant species is still unknown.

Four features of lncRNAs have been used to understand their conservation and evolution: primary sequences, transcription in syntenic regions, secondary structures, and function [17]. In terms of primary sequences of lncRNAs, only small stretches are conserved between homologous lncRNAs from different species. No homolog could be found for most vertebrate lncRNAs (>70%) in distant species (e.g. diverged >50 million years ago). Additionally, the exon-intron structure of lncRNAs changed quickly during the evolution of species [5]. However, lncRNAs and PCGs have comparably conserved promoters and TF binding sites, implying their potentially conserved transcriptional and epigenetic regulation [6]. Despite the rewired exon-intron structure of lncRNAs caused by the reshuffling of transposable elements (TEs), their 5’ and 3’ splicing sites seem to be well conserved [12,18,19]. Furthermore, two classes of conserved lncRNAs have been defined based on their selection signatures: one with purifying selection signals at the primary sequence, the other with the sign of selection for transcription [20]. Even though sequence similarity was only observed in a small section of homologous lncRNA sequences from different plant species, their functions could be well conserved. For example, the functionality of the photomorphogenesis-related lncRNA *HID1* was conserved between *Arabidopsis* and rice [4]. When transferred into the *hid1* mutant of *Arabidopsis, OsHID1* could restore the elongated hypocotyl phenotype of *hid1* [4]. Similar functional conservation has also been observed for other lncRNAs, such as *Xist* [21,22], *HOTAIR* [23,24], *MALAT1* [25–28], *IPS1* [9], and *ENOD40* [10,11]. The small patches of conserved sequences within lncRNAs are usually RNA binding sites of proteins (such as the RPC2 complex) that interact with lncRNAs and/or are critical for maintaining the secondary structure of lncRNAs [29]. Despite the low conservation of lncRNA sequences, the secondary structure of homologous lncRNAs is largely conserved across species. For example, the antisense lncRNA *COOLAIR* has the same function in the cold-induced flowering across diverse Brassicaceae species, including *Arabidopsis alpine*, one of the perennial relatives of *Arabidopsis thaliana* [30]. *COOLAIR* of the two species has the similar sophisticated secondary structures [31]. This implies the importance of secondary structure for the functionality of lncRNAs. In addition, for some lncRNAs, despite rapid changes of their sequences during evolution, their syntenic position was preserved, which could be used to identify homologous lncRNAs with divergent primary sequences, and such homologous lncRNAs have been identified in Brassicaceae and Cleomaceae [32]. Lastly, lncRNAs exhibit high turnover rates of transcription as demonstrated in mouse embryonic stem cells [20], nine different tissues from six mammals [33], mouse liver tissues [34], and several tissues from diverse Citrus species [35].

Despite large-scale comparisons of lncRNAs in animal genomes, the evolutionary dynamics and diversification of lncRNAs in plants are still unknown. The availability of high-quality plant genomes along with rich genomic resources, such as transcriptomes of different developmental stages in various plant species, allows us to identify lncRNAs and investigate the evolution of lncRNAs in plants [2]. LncRNAs consist of several large heterogeneous groups, including long intergenic non-coding RNAs (lincRNAs), intronic lncRNAs and antisense lncRNAs, in terms of their genomic positions. In this study, with a focus on lincRNAs, we identified thousands of lincRNAs in 26 plant species, including six non-flowering plants, analysed a homologous relationship of lincRNAs from different plant species based on their sequence conservation and syntenic relationship, and explored characteristics of lincRNAs that were conserved during the history of plant evolution. We found that most lincRNAs evolved rapidly in terms of both sequence and expression pattern and showed a high rate of transcriptional gain and loss. Our results also support TEs being the main source of lincRNAs that are under transcriptional regulation by histone modifications and transcription factors. Together, the results presented in this study provide a comprehensive view on the evolutionary landscape of plant lincRNAs.

## Results

### Genome-wide identification of lincRNAs in 26 plant species reveals conserved characteristics of lincRNAs

**Figure 1.**
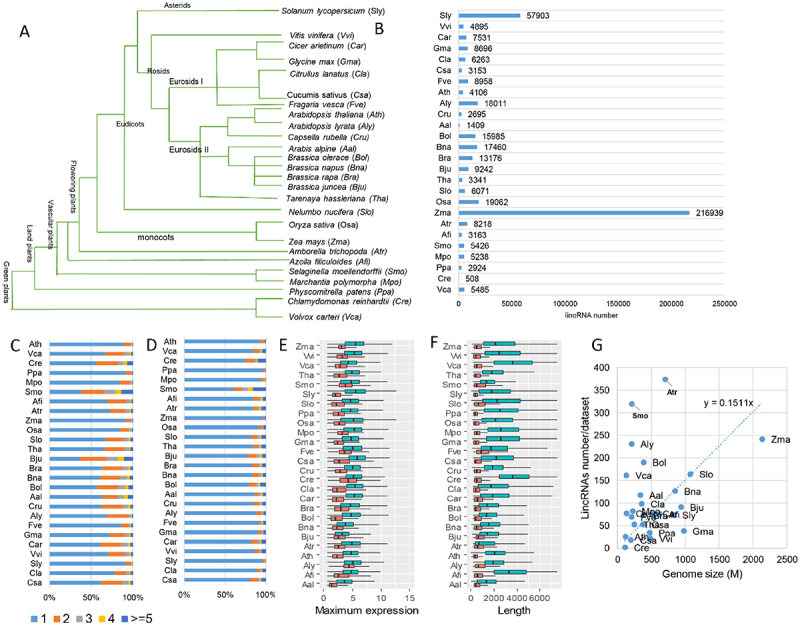
Genome-wide identification of lncRNAs across 26 plant species. (A) The phylogenetic tree of the 26 selected plant species, including six non-flowering plant species: *Cucumis sativus* (Csa), *Citrullus lanatus* (Cla), *Solanum lycopersicum* (Sly), *Vitis vinifera* (Vvi), *Cicer arietinum* (Car), *Glycine max* (Gma), *Fragaria vesca* (Fve), *Arabidopsis thaliana* (Ara), *Arabidopsis lyrata* (Aly), *Capsella rubella* (Cru), *Arabis alpine* (Aal), *Brassica oleracea* (Bol), *Brassica napus* (Bna), *Brassica rapa* (Bra), *Brassica juncea* (Bju), *Tarenaya hassleriana* (Tha), *Nelumbo nucifera* (Slo), *Oryza sativa* (Osa), *Zea mays* (Zma), *Amborella trichopoda* (Atr), *Azolla filiculoides* (Afi), *Selaginella moellendorffii* (Smo), *Marchantia polymorpha* (Mpo), *Physcomitrella patens* (Ppa), *Chlamydomonas reinhardtii* (Cre) and *Volvox carteri* (Vca). (B) The number of lincRNAs identified in each plant species. (C) The distribution of exon number of lincRNAs in each plant species. (D) The distribution of lincRNA isoform numbers in each plant species. (E) The maximum expression level of both lincRNAs and protein-coding genes (PCGs) in each plant species. (F) The genomic length of both lincRNAs and protein-coding genes (PCGs) in each plant species. (G) Correlation between genome size and the number of lincRNAs per sample identified.

To understand the evolution of plant lincRNAs and directly compare lincRNA transcripts from diverse plants, lincRNA transcripts were identified across 26 representative plant species, including six non-flowering plants (Fig. 1A). Harnessing the large number of RNA-seq datasets in each plant species (**Supplemental Table S1**), varying numbers of lincRNAs were identified (Fig. 1B). We observed a higher number of lincRNAs for plant species with larger genomes (e.g. *Zea mays*). Generally, the number of lincRNAs identified in the 26 plant genomes was roughly in a linear relationship with their genome size (**Figure S1D**), and the genome sizes were correlated with the number of lincRNAs identified (Fig. 1G). However, as demonstrated in other studies [5,6], direct comparison of lincRNA numbers in plant species was not easy because of the number and quality of the available RNA-seq data (**Figure S1A**), as well as the inherent heterogeneity in the sampled tissues. Differences in sequencing depth, variable genome size and assembly quality might contribute to the overall differences in the lncRNA numbers. And whether the variable size of the lincRNA repertoire found in different plant species is biologically meaningful (**Figure S1C**) is warranted for further investigation as the proportion of the expressed protein-coding genes (PCGs) in each plant was relatively uniform (**Figure S1B**). We found that most lincRNAs had a single exon and one isoform irrespective of the species studied (Fig. 1C, D). Furthermore, features such as the maximum expression level and the size of lincRNAs were largely consistent across plant species, including non-flowering plants (Fig. 1E, F), suggesting a comparable quality of the identified lincRNAs in different plant species. The expression levels of lincRNAs were consistently lower than that of PCGs (Fig. 1E). For the plants (e.g. rice and soybean) with publicly available lncRNAs, we also compared the lincRNAs identified in this study with the lncRNAs collected from publications and public databases (**Figure S1E**) and found that the majority of lincRNAs identified in this study were novel. It might be a result of using different criteria but might also imply the incompleteness of lincRNAs in plants because of limited samples used in each analysis that failed to capture all lincRNAs due to their spatiotemporal expression feature.

### Most lincRNAs are species-specific

**Figure 2.**
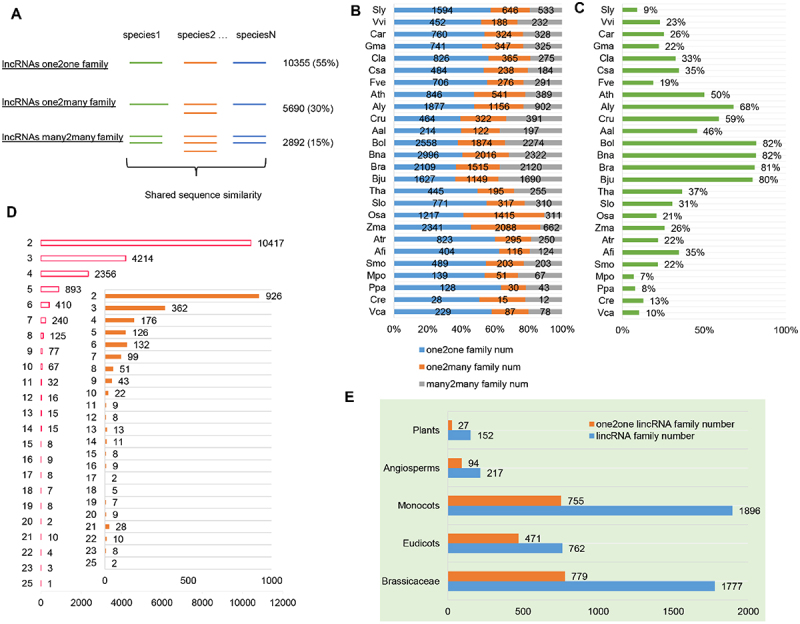
Conservation of lincRNAs by sequence similarity in plants. (A) Three types of lincRNA families based on sequence similarity: one2one family, one2many family and many2many family. The corresponding family number and percentage of each type are shown on the right of the graph. (B) The percentage of each type of lincRNA family in each plant species. (C) The percentage of homologous lincRNAs in each plant species. (D) The distribution of the number of lincRNA families shared within 2–25 species. Inset: The distribution of the number of *Arabidopsis thaliana* lincRNAs shared in 2–25 other species. (E) The number of conserved lncRNAs across different levels of evolutionary lineages in plants, including Plants, Angiosperms, Monocots, Eudicots and *Brassicaceae* (see M&M for definition).

Plant evolution has experienced several rounds of whole genome duplication (WGD), chromosome shuffle and local duplication, all of which could multiply the copy numbers of lincRNAs [36]. Additionally, many flowering plants, including many major crops, are polyploids [37]. To trace the conservation of homologous lincRNAs in different plant species, we grouped the lincRNAs based on three scenarios: only a single copy in each of the plant species in which the lincRNA was identified (defined as lincRNAs one2one family), a single copy in some plant species and multiple copies in others (defined as lincRNAs one2many family), and multiple copies in all plant species in which the lincRNAs were identified (defined as lincRNAs many2many family) (Fig. 2A). One typical example of each scenario is illustrated in **Supplemental Figure S2F**. The family relationships of the lincRNAs among the 26 plant species were established based on pairwise blast search and using the graph clustering method MCL. As a result, the lincRNAs were classified into 18,937 families, including 10,355 (55%) one2one families, 5,690 (30%) one2many families, and 2,892 (15%) many2many families (Fig. 2A**, Supplemental Table S2**). Over half of the lincRNA families were classified as one2one families, possibly suggesting rapid evolution of lincRNA loci. In the six non-flowering plants, including *Azolla filiculoides* (Afi),*Selaginella moellendorffii* (Smo),*Marchantia polymorpha* (Mpo),*Physcomitrella patens* (Ppa), *Chlamydomonas reinhardtii* (Cre) and *Volvox carteri* (Vca), 2003 (11%) lincRNA families representing the most ancient ones were found.

Homologous lincRNAs identified in different plant species usually shared only short patches (~60 nt) of sequence conservation (**Figure S2A**) with <10 mismatches within each patch (**Figure S2B**). Furthermore, a significant number of the lincRNAs identified here overlapped with the conserved non-coding sequences (CNSs) reported in several previous studies (**Figure S2C, D, E**) [38–40]. In each plant species, the one2one family was the dominant one (Fig. 2B). Most plant species except the Brassicaceae family had a low (<50%) percentage of homologous lincRNAs (Fig. 2C), implying that most lincRNAs were species-specific due to rapid gain and/or loss during plant evolution, which is evident and demonstrated in the distribution of the number of homologous lincRNAs found in different species (Fig. 2D). For example, in *Arabidopsis thaliana* (Ath), only 476 (476/4106, 11.6%) lincRNAs of 313 families (257 many2many, 38 one2many, 18 one2one lincRNA families) were highly conserved (defined as with homologous lincRNAs in at least six species) (Fig. 2D). Intriguingly, of the 476 highly conserved Ath lncRNAs, many of them were flanked by PCGs related to flowering and/or flower development, such as *FLO5, UFO1* and *SACS3*, which presumably implies that these highly conserved lncRNA families may also be implicated in biological processes related to flower development. Although lincRNAs displayed rapid sequence divergence compared to PCGs, 217 lincRNA families (94 one2one families) identified in flowering plants (Angiosperms) had detectable sequence conservation, and they made up a small subset of the lincRNAs that emerged over the past ~200 million years of flowering plant evolution (Fig. 2E**, Figure S3A, B**). Together, identification and characterization of lincRNA families across the whole plant lineages revealed a rapid evolution of primary sequences of lincRNAs, but still a small portion of lincRNAs was well preserved during the evolution history of flowering plants.

### Transcriptional regulation of ancient lincRNAs in plants

**Figure 3.**
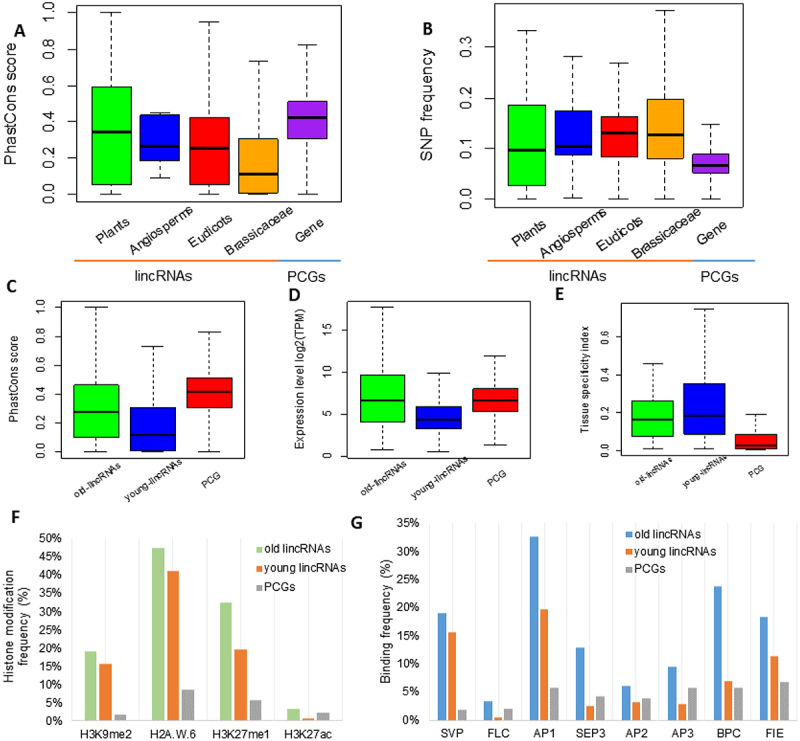
Active regulation of ancient lincRNAs in plants. (A) Sequence conservation (PhastCons Score) of the lincRNAs among the 20 flowering plants at four levels of evolutionary age (Plants, n = 71; Angiosperms, n = 11; Eudicots, n = 65; Brassicaceae, n = 556). Protein-coding genes (PCGs) (Gene, n = 27,655) were used as control. (B) SNP frequency (SNPs/100-bp) in lincRNAs from different evolutionary age classes (Plants, n = 71; Angiosperms, n = 11; Eudicots, n = 65; Brassicaceae, n = 556). PCGs (Gene, n = 27,655) were used as control. (C) Sequence conservation (PhastCons Score) of the old and young lincRNAs from the 20 flowering plant species. Old lincRNAs (n = 148): lincRNAs of the evolutionary age classes of Plants, Angiosperms and Eudicots; young lincRNAs (n = 566): lincRNAs of the evolutionary age classes of Brassicaceae. PCGs (Gene, n = 27,655) were used as control. (D) The expression level of the old and young lincRNAs. (E) Tissue specificity index of the old and young lincRNAs. (F) Frequency of histone modification (H3K9me2, H2A.W.6, H3K27me1 and H3K27ac) in 1-kb upstream/downstream regions of the old lincRNAs, young lincRNAs and PCGs. (G) Frequency of binding sites for transcriptional factors (SVP, FLC, AP1, AP2, AP3, BPC, SEP3 and FIE) in 1-kb upstream/downstream regions of the old lincRNAs, young lincRNAs and PCGs.

Fast lincRNA evolution prohibits the identification of lincRNA homologs in distant species using the sequence homology-based approach. It contributes to a smaller proportion of conserved lincRNAs in plants. In order to further understand the conservation of lincRNAs and the regulatory mechanism(s) conferred by the conserved lincRNAs, we compared the PhastCons scores of lincRNAs within different evolutionary age groups (EAGs) with that of PCGs. PhastCons scores were calculated based on DNA sequence conservation metrics of 20 angiosperm plant genomes [41] and the 1001 *Arabidopsis* genomes datasets [42]. Four EAGs determined based on the number of lincRNAs homologous to those of *A. thaliana* were used in the comparison. They were Plants (n = 71), Angiosperms (n = 11), Eudicots (n = 65) and Brassicaceae (n = 556). The PhastCons scores decreased from the EAG Plants to the EAG Brassicaceae and the median conservation score of lincRNA in the EAG Plants (~0.34) was comparable with that of PCGs (~0.42) (Fig. 3A**, Figure S4A**).

The frequency of single nucleotide polymorphisms (SNPs/100-bp) increased from the EAG Plants to the EAG Brassicaceae, but their SNP frequencies were all higher than that of PCGs (Fig. 3B**, Figure S4F**), suggesting that potential purifying selection and relaxation of constraints may be the driving force responsible for the lower conservation of lincRNAs. Conservation of the upstream and downstream regions of lincRNAs was comparable with that of PCGs in both *Arabidopsis* and rice (**Figure S4B, C, D, E**). Furthermore, the more conserved old lincRNAs (defined as those in the EAGs of Plants, Angiosperms and Eudicots; Fig. 3C) seemed to have higher expression levels (Fig. 3D) and lower tissue specificity (Fig. 3E) compared to that of young lincRNAs (defined as those in the EAG of Brassicaceae). Besides, the comparable expression levels between the old lincRNAs and PCGs imply conserved evolutionary selective pressure for these two groups of transcripts at the levels of transcriptional and chromatin regulation.

We observed comparable frequencies of histone modifications and transcription factor (TF) binding sites in regions of lincRNAs, suggesting transcriptional regulatory role of lincRNAs (**Figure S3C, D**). Interestingly, several histone modifications (e.g. H3K9me2, H3K27me1, H2A.W.6 and H3K27ac) and MADS TFs (the master regulators of flower development) were found to preferentially bind to the regulatory regions (1-kb upstream/downstream) of lincRNAs in plants (Fig. 3F, G**; Figure S4G, H**), while, a different set of histone modifications (e.g. H3K36me2, H3K4me1, H3K4me2, H3K36me3, H3K18ac, H3K4me3, and H3K9ac) and TFs were found to be preferentially associated with PCGs (**Figure S3E, F**). For example, ~13% of old lincRNAs contained the SEP3 binding sites, five times higher than the same binding sites observed in PCGs (~2.5%) in *A. thaliana*. The higher association of old lincRNAs with MADS TFs implies important functions of the old lincRNAs in flower development. Supporting this notion, a genome-wide study of ancient lncRNAs in tetrapods has found that the association between old lincRNAs and homeobox TFs plays a role in embryonic development [6]. The high enrichment of H3K9me2, H3K27me1 and H2A.W.6 in old lincRNAs suggests the association of conserved lincRNAs with heterochromatin regions enriched with transposable elements (TEs) (Fig. 3F); in contrast, PCGs seemed to be more associated with transcriptional chromatin environment (**Figure S3F**). Taken together, these results demonstrate tight regulation of the highly conserved old/ancient lincRNAs by TFs or TEs.

### The expression pattern of lincRNAs suggests their high rate of transcriptional turnover

**Figure 4.**
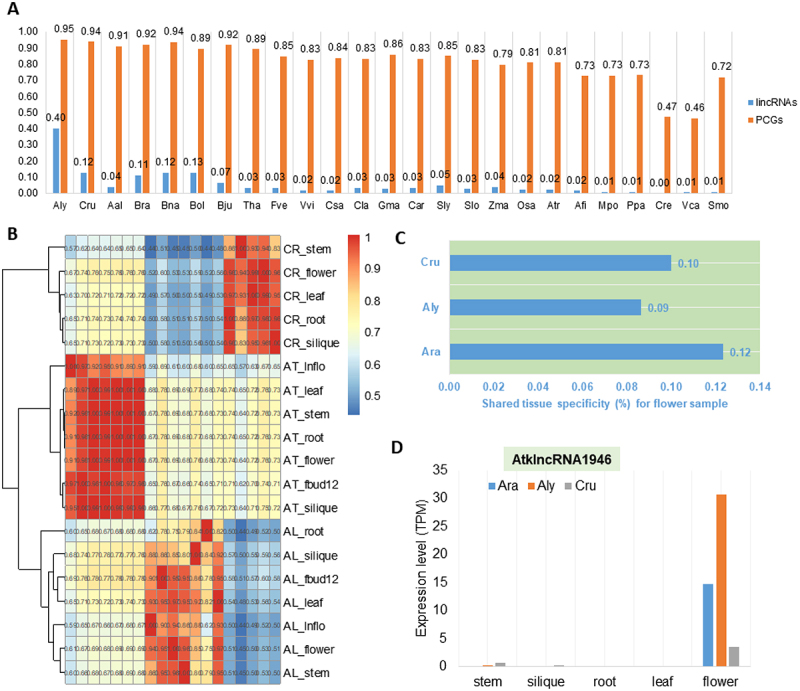
The rapid transcriptional turnover of lincRNAs during the evolution of *Arabidopsis thaliana, Arabidopsis lyrata* and *Capsella rubella*. (A) Percentage of *A. thaliana* lincRNAs and protein-coding genes transcribed in other 25 plant genomes, including the six non-flowering plants. (B) Hierarchical clustering of pairwise correlations of the lincRNA families in *A. thaliana, A. lyrata* and *C. rubella*. AT_: tissues of *A. thaliana*; AL_: tissues of *A. lyrata*; CR_: tissues of *C. rubella*. (C) The proportion of lincRNAs sharing flower expression specificity in *A. thaliana, A. lyrata* and *C. rubella*. (D) The expression level of *AtklncRNA1946* in different tissues of *A. thaliana, A. lyrata* and *C. rubella.*

In order to estimate the transcriptional activity of conserved lincRNAs across diverse plant species, we investigated transcription of *A. thaliana* lincRNAs and PCGs (used as a control) in other 25 plant species. We found that transcription of lincRNAs homologous to those of *A. thaliana* was only evident in the plant species that are closely related to *A. thaliana* (Fig. 4A). For example, about 40% of *A. thaliana* lincRNAs showed transcription in *A. lyrata,* while only ~1% of *A. thaliana* lincRNAs showed transcription in non-flower species. It is clear that even for *A. lyrate*, in which the highest transcription percentage of lincRNAs homologous to those of *A. thaliana* was observed, the percentage was less than half of the transcription percentage of PCGs, whereas PCGs of *A. thaliana* were relatively constantly transcribed in other plant species (Fig. 4A). These results suggest that the lincRNA expression pattern evolved quickly. To investigate the possible influence of tissues and samples on the results, we compared tissue specificity for the expressed lincRNAs in the three representative species of Brassicaceae: *A. thaliana, A. lyrata* (Aly) and Capsella *rubella* (Cru), for which data from equivalent tissues were available. When the hierarchical clustering method was used to cluster the expression of lincRNAs from different samples of the three species, it was clear that different tissues from the same species were always clustered together (Fig. 4B). However, only 12% of *A. thaliana* lincRNAs expressed in flower shared tissue specificity with the other two species (Fig. 4C); similarly, ≤10% of Aly and Cru flower lincRNAs had their homologs expressed in Ath flowers, suggesting a significant tissue specificity of flower lincRNAs (P < 0.01). The lincRNAs universally expressed in the flower tissues but not in other tissues in these three plant species would have a conserved function in flower development, such an example (*AtklncRNA1946*) is shown in Fig. 4D.

**Figure 5.**
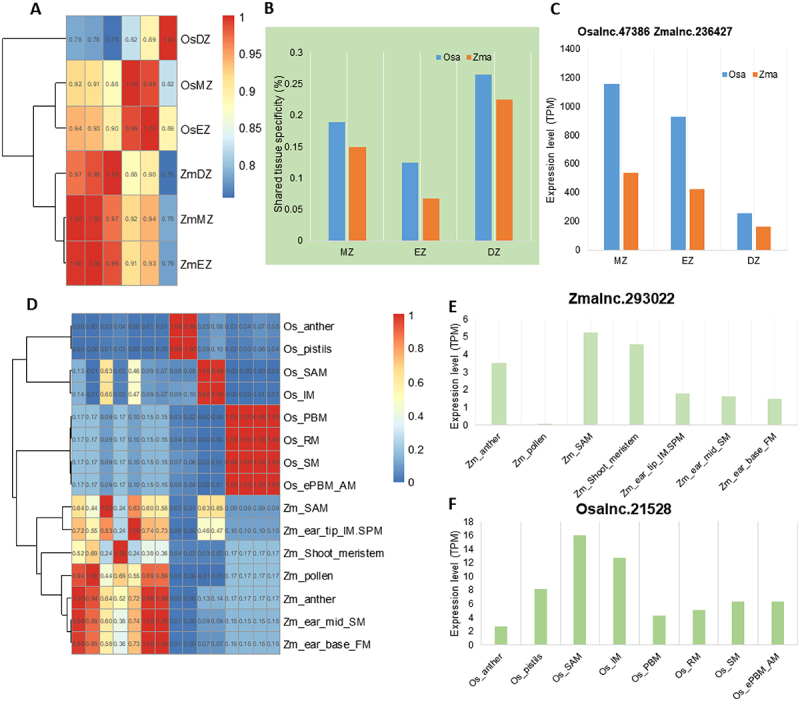
The rapid transcriptional turnover of lincRNAs during the evolution of *Oryza sativa* and *Zea mays*. (A) Hierarchical clustering of pairwise correlations of lincRNA families in *O. sativa* and *Z. mays*. Os_: tissues of *O. sativa*; Zm_: tissues of *Z. mays*. (B) The proportion of lincRNAs sharing flower expression specificity in *O. sativa* and *Z. mays*. MZ: root meristematic zone; EZ: root elongation zone; DZ: root differentiation zone. (C) Conserved expression of *Osalnc.47386* and *Zmalnc.236427* in *O. sativa* and *Z. mays*. (D) Hierarchical clustering of pairwise correlations of lincRNA families during the evolution of *O. sativa* and *Z. mays*. Os_: tissues of *O. sativa*; Zm_: tissues of *Z. mays*. (E) The expression level of Zmalnc.293022 in different tissues of *Z. mays*. (F) The expression level of Osalnc.21528 in different tissues of *O. sativa*. (E) and (F) show the conserved expression of the homologous pair lincRNAs in SAM.

To understand whether similar tissue-specific expression of lincRNAs is applied in monocots, we further investigated transcriptional profiles of lincRNAs in two representative monocot species, *Oryza sativa* and *Z. mays*, using RNA-seq datasets generated from different zones of elongating roots. Samples from the same species were grouped together (Fig. 5A), and a relatively small percentage (<25%) of lincRNAs shared tissue specificity (Fig. 5B). For example, only 18% of *O. sativa* lincRNAs shared tissue specificity with *Z. mays* in the root meristematic zone. Nevertheless, some lincRNAs that were expressed in roots of both rice and maize, their expression patterns were quite constant in the two species, implying they may have a conserved function in root development. One such lincRNA is the pair *Osalnc.47386* and *Zmalnc.236427* that showed a decreasing expression pattern from root tip to differential zone (Fig. 5C). A similar situation was evident for lincRNAs found in flower and reproductive tissues. LincRNAs were preferentially grouped together according to their origin, i.e. *O. sativa* or *Z. mays* (Fig. 5D). But, like the conserved lincRNAs found in roots, lincRNAs expressed in specific tissues of both rice and maize were also found, such as the pair *Zmalnc.293022* and *Osalnc.21528* found in the shoot apical meristem (Fig. 5E, F).

### Sequence-based homologous lincRNAs are largely not overlapping with synteny-based ones

**Figure 6.**
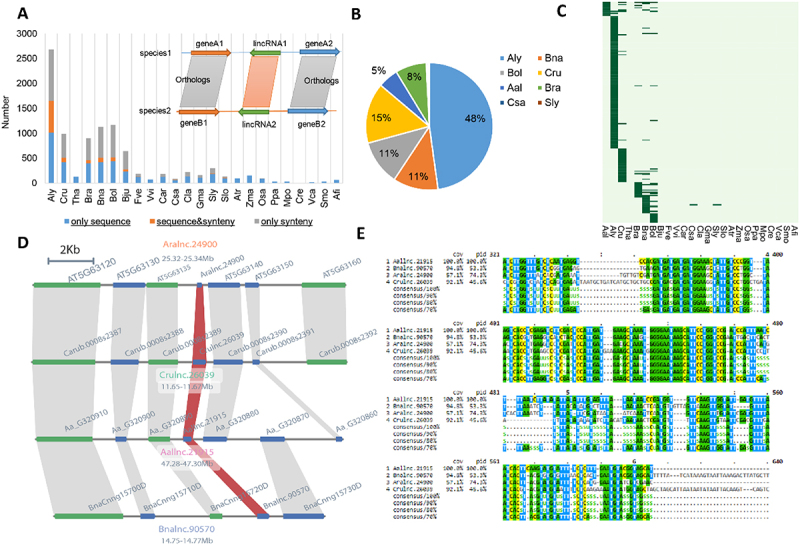
Conservation of lincRNAs in *Brassicaceae* family. (A) Number of *Arabidopsis thaliana* lincRNAs with different levels of homologous (sequence only, sequence&synteny and synteny only) in other species. Inset: a diagram showing syntenic homologous lincRNAs. (B) Distribution of the 199 *A. thaliana* lincRNAs with sequence&synteny homologs in other eight plant species. Most *A. thaliana* lincRNAs have homologs in the *Brassicaceae* family, especially in the closely related *A. lyrate*, and just few in Csa and Sly (not showing in the pie graph). (C) Conservation of the 199 *A. thaliana* sequence&synteny lncRNAs. Each horizontal bar represents a homologous lincRNA in the corresponding species shown on the X-axis. (D) Syntenic relationship between *Aralnc.24900* and its homologs on other plant species. (E) Sequence alignment of *A. thaliana Aralnc.24900* and its homologs from *Arabis alpine* (*Aallnc.21915), Capsella rubella* (*Crulnc.26039*) and *Brassica napus* (*Bnalnc.90570*).

Many putative lincRNA homologs cannot be detected through sequence similarity owing to their rapid sequence divergence; however, the genomic positions of such lincRNAs could be conserved during plant evolution. Therefore, the syntenic relationship of highly conserved PCGs could be used for identified lincRNAs flanking the syntenic PCGs despite little sequence similarity between the potentially homologous lincRNAs (Fig. 6A). Here, a syntenic block was defined when one or more of the three PCGs on each side of a given lincRNA and a total of three or more PCGs showed syntenic relationship [43]. Using lincRNAs from *A. thaliana* as references, hundreds of syntenic lincRNAs were detected in other species of the Brassicaceae family and the number of synthenic lincRNAs dramatically reduced in plants that are evolutionarily distant from *A. thaliana*. A small portion of lincRNAs showed both sequence and synteny homologys (Fig. 6A). For example, in *A. lyrata*, 1592 lincRNAs were identified based on sequence homology, of which only 121 lincRNAs were detected by the synteny-based method. Similar results were observed when using lincRNAs from other plants (e.g. *A. lyrata*) as the reference (**Figure S5A**). Additionally, when using lincRNAs from other plants as the reference, the vast majority of lincRNAs identified based on the sequence similarity approach could not be identified based on syntenic relationship in the distant species (**Figure S5**).

Between the two monocots (rice and maize), most lincRNAs only shared syntenic homology or sequence homology (**Figure S5C, D**). When considering the distribution of *A. thaliana* homologous lincRNAs detectable by both the sequence and synteny-based approaches, we found that most of them were shared in the Brassicaceae family, especially in its nearest relative *A. lyrata* (Fig. 6B). Among the 199 *A. thaliana* lincRNAs with both sequence- and synteny-based homologs identified in other plant species, most were found to be conserved only in a single species (Fig. 6C). LincRNAs supported by both sequence and synteny-based homologys were highly conserved in the Brassicaceae family. We found at least 34 such lincRNAs (**Figure S6**), and one example is illustrated in Fig. 6D.

### Synteny- and gene network-based functional characterization of conserved lincRNAs

LincRNAs regulate gene expression by in *cis* or in *trans* mechanism [44]. To investigate potential functions of lincRNAs, we used neighbouring PCGs to infer in *cis* functions of lincRNAs and used gene co-expression relationship to infer their potential in *trans* functions. Some lincRNAs were flanked by conserved PCGs related to flowering pathways, such as *BRC1/TB1* (**Figure S6, Figure S7A**), *AG* (**Figure S7B**), *LFY* (**Figure S7C**), *SEP1* (**Figure S7D**), *FT/ZCN* (**Figure S7E**), and *SOC1* (**Figure S7F**), suggesting that these lincRNAs may potentially function in *cis* to regulate the conserved functions of their neighbouring PCGs.

**Figure 7.**
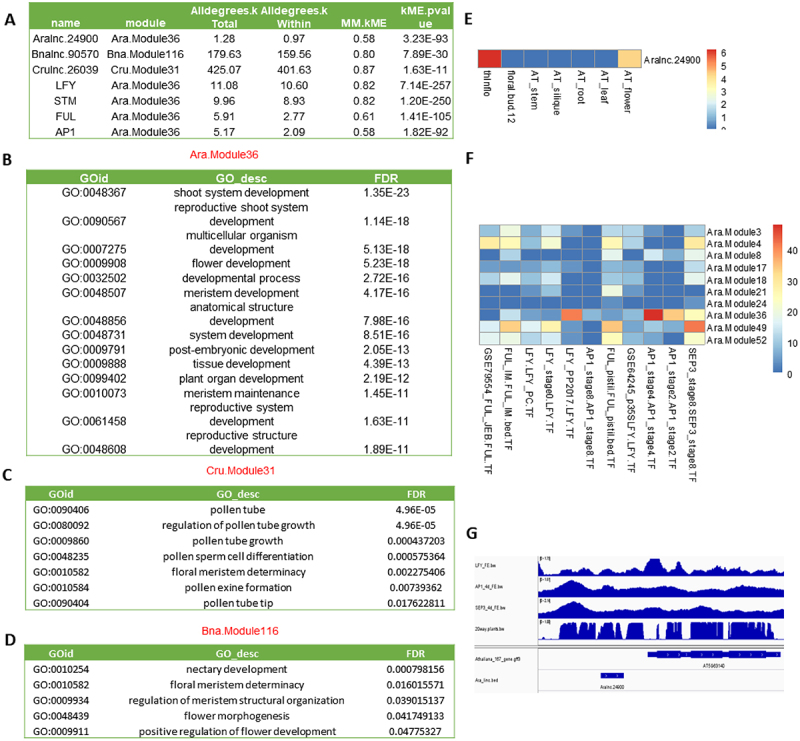
The functionality of *Aralnc.24900* and its homologs based on co-expression analysis. (A) Characteristics of *Aralnc.24900* and its homologs in Ban and Cru as well as the transcription factors interacting with *Aralnc.24900*. (B) GO annotation of the genes co-expressed with *Aralnc.24900* in Ara.Module36 in *Arabidopsis thaliana*. (C) GO annotation of the genes co-expressed with Crulnc.26039 in the module of Cru.Module31 in *Capsella rubella*. (D) GO annotation of the genes co-expressed with *Bnalnc.90570* in the module of Bna.Module116 in *Brassica napus*. (E) The expression pattern of *Aralnc.24900* in different tissues of *A. thaliana*. (F) The enrichment of transcription factors (AP1, SEP3, LFY and FUL) interacting with *Aralnc.24900* in different flower-related co-expression modules. (G) The coverage map showing *Aralnc.24900* bound by LFY, AP1 and SEP3 TFs in *A. thaliana*. 20way.plants.bw: the track of PhastCons scores.

We used the expression levels of PCGs and lincRNAs within multiple samples to compute their co-expression relationship using WGCNA in the following seven representative plant species: *A. thaliana, A. lyrata, C. rubella, B. napus, O. sativa, Z. mays* and *M. polymorpha*. In each plant species, several co-expression modules were identified and the PCGs included in each module were subjected to GO enrichment analysis. Here, we presented the flowering-related modules to illustrate the results. The PCGs within the module Ara.Module36 were enriched with GO terms related to flower development (qvalue = 1.91e-20), meristem development (qvalue = 1.60e-18) and meristem maintenance (qvalue = 7.54e-14), and these PCGs had strong expression levels in meristems and flowers (**Figure S8A**). We hypothesized that the lincRNAs within each of these modules would function in the same pathway(s) as their co-expressed PCGs. For instance, in the module of Ara.Module36, an lincRNA, *Aralnc.24900*, well conserved in Aal (*Aallnc.21915*), Bna (*Bnalnc.90570*) and Cru (*Crulnc.26039*) (Fig. 6D), was co-expressed with several flowering-related genes, including *LFY, STM, FUL* and *AP1* (**Figure S8B**, Fig. 7A, B). Aligning these lincRNA sequences found many conserved motifs that may potentially serve as RNA binding sites or other functionality (Fig. 6E). Indeed, some of those were binding sites of several master regulatory TFs (e.g. *LFY, AP1* and *SEP3*) of flower development (Fig. 7F, G). Additionally, the homologous lincRNAs of *Aralnc.24900* were also found in flower-related modules in Cru (Cru.Module31) (Fig. 7A, C) and Bna (Bna.Module116) (Fig. 7A, D), in which they were co-expressed with the same sets of flower-related genes identified in Ara.Module36. Moreover, these homologous lincRNAs had similar expression patterns in inflorescence meristems and flowers (Fig. 7D, E, G).

**Figure 8.**
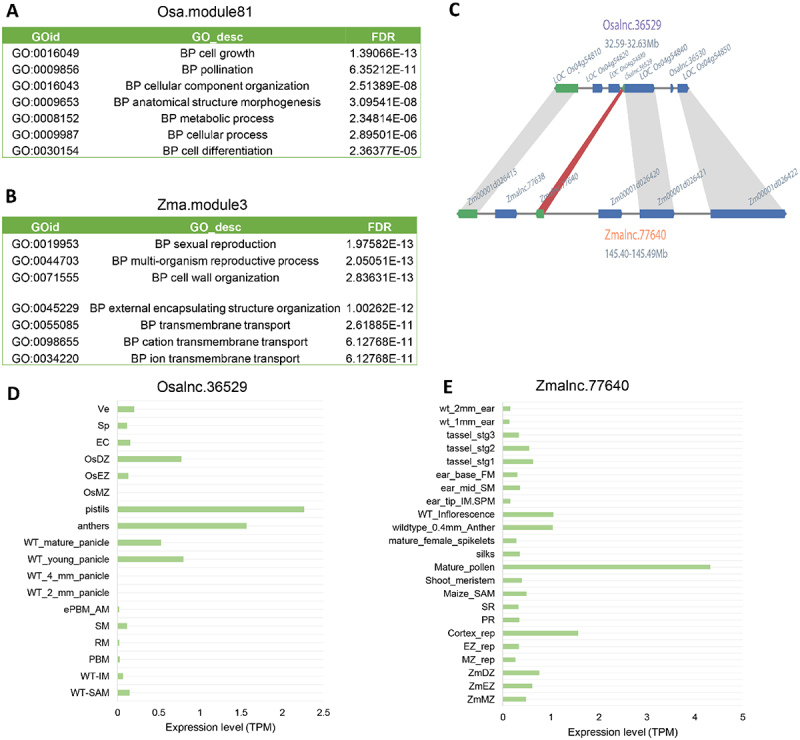
Conservation of lincRNAs in the grass family. (A) GO annotation of the genes co-expressed with *Osalnc.36529* in the flower-related Osa.module81. (B) GO annotation of the genes co-expressed with *Zmalnc.77640* in the flower-related Zma.module3. (C) The syntenic relationship between *Osalnc.36529* and *Zmalnc.77640*. (D) The expression pattern of *Osalnc.36529* in different tissues of *Oryza sativa*. (E) The expression pattern of *Zmalnc.77640* in different tissues of *Zea mays.*

We also investigated the potential functions of the lincRNAs conserved in *O. sativa* and *Z. mays* (Fig. 8). Based on sequence similarity, 235 and 2879 lincRNAs were identified in *O. sativa* and *Z. mays*, respectively. Of these conserved lincRNAs, seven were also identified based on the synteny homology approach. In order to infer the functionality of these conserved lincRNAs, for each species, co-expression networks involving lincRNAs and PCGs were constructed by WGCNA, from which several co-expression modules were identified. Similar to the results achieved in dicots, we also found a co-expression module enriched with PCGs related to flower/meristem development in both rice and maize (**Figure S9A, B, S10**), implying similar functions of the lincRNAs and PCGs of the corresponding modules identified in the two plant species (**Figure S9A, B, S10**). For instance, *Osalnc.36529* of *O. sativa* and *Zmalnc.77640* of *Z. mays* were found in the syntenic region (Fig. 8C), and in the flower-related module Osa.module81 (Fig. 8A) and Zma.module3 (Fig. 8B), respectively. Osa.module81 was enriched with GO:0016049 (cell growth, qvalue = 1.39E-13), GO:0009856 (pollination, qvalue = 6.35E-11) and GO:0030154 (cell differentiation, qvalue = 0.0000236) (Fig. 8A), and *Osalnc.36529* was highly expressed in anthers and pistils (Fig. 8D). Correspondingly, Zma.module3 had functions related to GO:0019953 (sexual reproduction, qvalue = 1.98E-13), GO:0044703 (multi-organism reproductive process, qvalue = 2.05E-13) and GO:0071555 (cell wall organization, qvalue = 2.84E-13) (Fig. 8B), and *Zmalnc.77640* was highly expressed in pollens (Fig. 8E). Based on these results, we conclude that highly conserved lincRNAs may have similar functionality in different plants and act coordinately with their co-expressed PCG partners.

### TEs driving evolutionary stabilization of lincRNAs in plants

**Figure 9.**
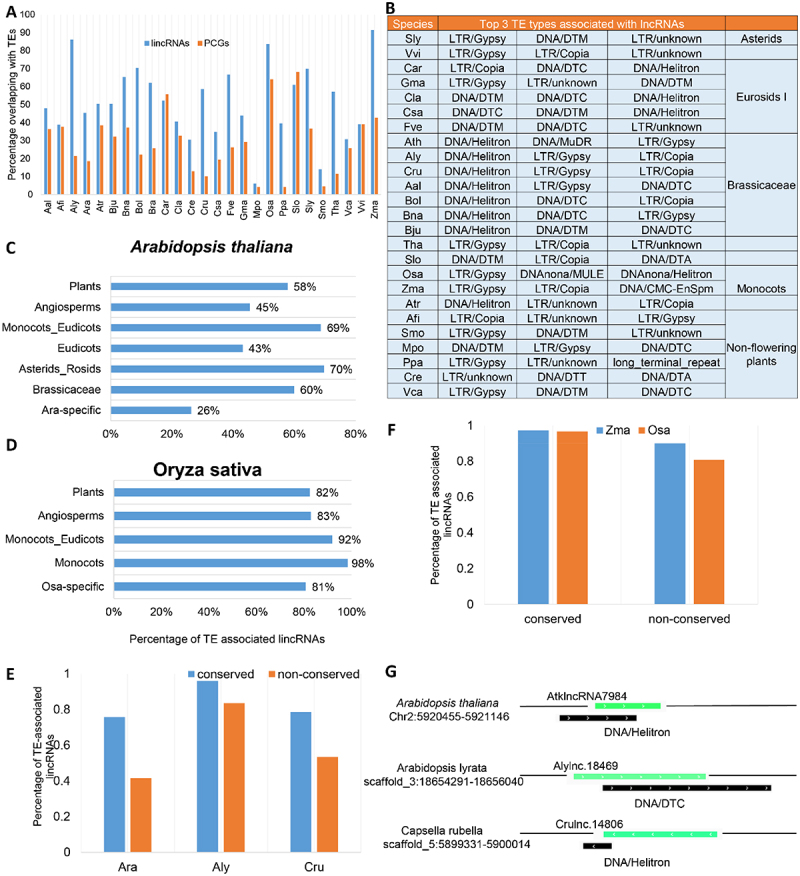
Transposable elements (TEs) drive the evolutionary origins of lincRNAs. (A) Fraction of lincRNAs and protein-coding genes overlapping with TEs. (B)Top three TE types in terms of percentage of lincRNAs overlapping with TEs in different plant species. (C) The percentage of *Arabidopsis thaliana* lincRNAs overlapping with TEs in different evolutionary age groups (in decreasing order; Plants: n = 71; Angiosperms: n = 11; Monocots_Eudicots: n = 242; Eudicots: n = 65; Asterids_Rosids: n = 135; Brassicaceae: n = 556; Ara-specific: n = 2044). (D) The percentage of *Oryza sativa* lincRNAs overlapping with TEs in different evolutionary age groups (in decreasing order; Plants: n = 262; Angiosperms: n = 111; Monocots_Eudicots: n = 1023; Monocots: n = 2482; Osa-specific: n = 15,073). (E) Comparison of the conserved and non-conserved lincRNAs overlapping with TEs in *A. thaliana* (Ath), *A. lyrata* (Aly) and *Capsella rubella* (Cru). (F) Percentage of the conserved and non-conserved lincRNAs overlapping with TEs in *O. sativa* and *Zea mays*. (G) Schematic representation of the overlapping between lincRNAs and TEs in *A. thaliana* (Ath), *A. lyrata* (Aly) and *C. rubella* (Cru). Green bars represent lincRNAs, and black bars are TEs.

A large portion of lincRNAs have been found to be overlapping with transposable elements (TEs) in human and mouse [18]. The number of TEs varies significantly in different plant species, and many plant genomes such as *Z. mays* possess high contents of TEs. TEs have been demonstrated to have significant impacts on plant genome evolution. But how about their role in the evolution of plant lincRNAs? To address this question, we checked the overlapping between the identified lincRNAs and TEs in each plant genome. We found a higher association between lincRNAs and TEs than between PCGs and lincRNAs (Fig. 9A). The highest association between lincRNAs and TEs was found in maize, but interestingly *A. lyrata* (Aly) had a higher rate of association (86.2%) than most of other plants despite its modest TE content (~31.1%) in the genome. LincRNAs associated with TEs have been suggested to be rewired by the associated TEs [45]. We found that lincRNAs of different plant families were linked with different types of TEs (Fig. 9B). For example, in the Brassicaceae family, DNA/Helitron seemed to be the dominant ones; however, in monocots, the dominant TEs were LTR/Gypsy.

In order to understand whether and how TEs contributed to lincRNA evolution in plants. We compared the percentage of the conserved lincRNAs associated with TEs in the representative eudicot (Ath) and monocot (Osa) species, and in different evolutionary age groups of the species. In *A. thaliana*, species-specific (Ath-specific) lincRNAs were depleted of TEs (Fig. 9C) compared with those conserved in different evolutionary age groups, while in *O. sativa*, the proportion of species-specific lincRNAs associated with TEs was similar to that observed in other evolutionary age groups (Fig. 9D). The lincRNAs conserved in rice were more likely to be associated with TEs than those conserved in *A. thaliana*, a phenomenon might be related to the transposition mechanisms of the TEs involved (retrotransposons vs transposons) (Fig. 9C, D). We further used lincRNAs conserved in Ath, Aly and Cru, and the remaining lincRNA in each of the three species to compare their association with TEs. No matter which species, Ath, Aly or Cru, conserved lincRNAs always had a higher fraction associated with TEs compared to the non-conserved ones (Fig. 9E). An example of an lincRNA conserved in Ath, Aly and Cru and their associated TEs is illustrated Fig. 9G. Similar result was also observed in the monocot species *O. sativa* and *Z. mays* although with a less difference (Fig. 9F). In summary, compared to PCGs, lincRNAs are more likely to be associated with TEs than PCGs but with different association features in different evolutionary lineages, which may be the driving force for the evolution of lincRNAs or the origin of lincRNAs.

### LincRNAs in non-flowering plants

**Figure 10.**
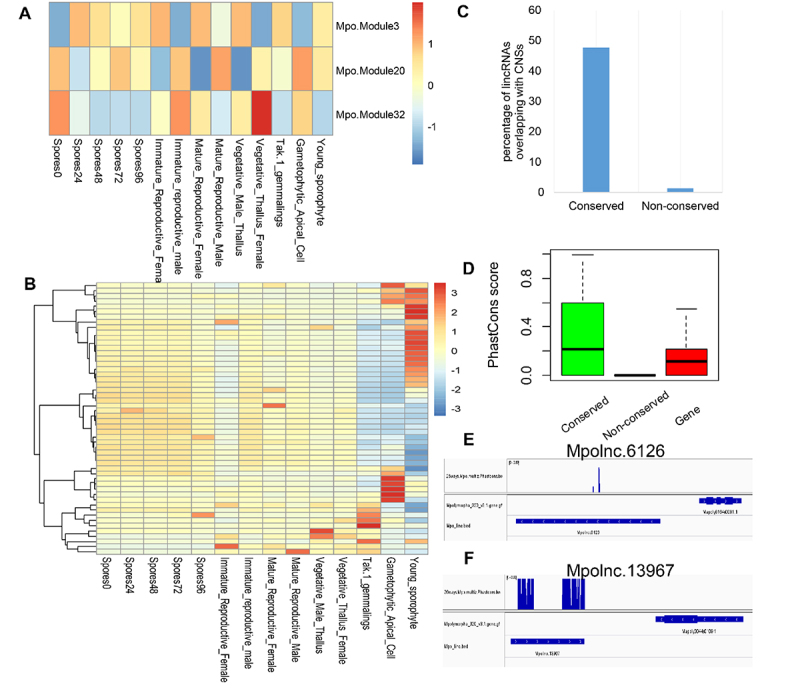
**LincRNAs in the land plant *Marchantia polymorpha***. (A) Expression pattern (eigen genes in each module) of meristem-related modules in different tissues of *Marchantia polymorpha*. The co-expression modules involving protein-coding genes (PCGs) and lincRNAs in the land plant *Marchantia polymorpha* was generated by WGCNA. (B) The expression pattern of the lincRNAs conserved in the three meristem-related modules (Mpo.Module3, 30, 32) across different tissues. (C) Conserved lincRNAs in *Marchantia polymorpha* are enriched in conserved non-coding sequences (CNSs). (D) Sequence conservation (PhastCons Score based on 26 plant genomes) of conserved lincRNAs, non-conserved lincRNAs and PCGs in *Marchantia polymorpha*. (E) PhastCons Score of *Mpolnc.6126* (in Mpo.Module32). (E) PhastCons Score of the *Mpolnc.13967* (in Mpo.Module3).

Based on the direct comparison of lincRNA sequences from each of the three non-flowering plants (the model alga *Chlamydomonas reinhardtii*, the model land plants *Physcomitrella patens* and *Marchantia polymorpha*) to those from other plant species, 65 (12.8%), 232 (7.9%) and 369 (7.0%) conserved lincRNAs were found in *C. reinhardtii, P. patens* and *M. polymorpha*, respectively. However, none of these sequence-based conserved lincRNAs could be detected by synteny-based approach, probably, because of disruption of the syntenic blocks during the long history of plant evolution. Nevertheless, the existence of sequence-based homologous lincRNAs in non-flowering plants suggests that they may have potentially conserved function(s). Based on co-expression network analysis, several meristem development-related modules (e.g. Mpo.Module3, 20, 32) were identified in *M. polymorpha*, and some lincRNAs of these three modules were highly expressed in reproductive tissues and sporophytes and potentially linked with flower and anther development (Fig. 10A, B). These lincRNAs may represent the most ancient functional lincRNAs. Furthermore, conserved lincRNAs in *M. polymorpha* were linked with conserved non-coding sequences (CNSs) (Fig. 10C) and had a higher PhastCons score than the non-conserved ones (Fig. 10D). Two conserved lincRNAs within the meristem-related modules are illustrated in Fig. 10E, F.

## Discussion

Genome-wide investigation of lincRNAs in 26 plant genomes provides clues about the evolution and conserved features of lincRNAs in plants. Direct comparison of lincRNAs among plant species reveals that most lincRNAs are species-specific, suggesting rapid evolution of lincRNAs in plants. The expression pattern of lincRNAs suggests their high rate of transcriptional turnover. Moreover, conserved lincRNAs show transcriptional regulation by transcriptional factors, such as AP1 and SEP3, which have a conserved functionality related to flower development in both the Brassicaceae and grass families. Furthermore, TEs are frequently associated with lincRNAs and drive stabilization of lincRNAs during the evolution of plants.

### The plant lincRNA repertory is far from completeness

Even though a large number of lincRNAs have been identified in diverse plants, thousands of new lincRNAs were still identified in this study by using publicly available RNA-seq datasets (**Figure S1**) presumably due to their high tissue specificity and low expression levels. For example, only 12% of Ath lincRNAs and 26% of Gma lincRNAs identified in this study overlap with the previously found ones [46,47]. Inter-individual variations in the same plant species are challenging for the comprehensive annotation of lincRNAs in plants. This was not explored in this study but has been shown in humans that lincRNAs of primary granulocytes exhibited higher expression variability in different individuals [48]. Furthermore, lincRNAs are heterogeneous groups of RNAs, present in different forms (e.g. linear or circular), possessing diverse properties (e.g. with or without polyA tail) and showing variable stability (i.e. stable or unstable). CircRNAs are closed RNAs formed by back-joining of splicing acceptor and donor. CircRNAs and lincRNAs without polyA tails cannot be efficiently identified using the RNA-seq datasets used in this study. Some types of lincRNAs such as promoter upstream transcripts (PROMPTs) are unstable and rapidly degraded by nuclear RNA decay pathways and thereby can only be seen in mutants of components in the exosome [49]. Finally, the predicted gene model of lincRNAs is prone to be inaccurate and fragmented due to the limitation of Illumina short RNA-seq reads and thereby molecular techniques such as RACE would be needed for verification. The third generation of sequencing technology such as PacBio/SMRT and Nanopore that can directly sequence the full length of lincRNAs, as demonstrated in studies of human lincRNAs [50], would be the ideal tool for identification of full-length lincRNAs in plants.

### The conserved evolutionary landscape of lincRNAs across eukaryotic species

The evolutionary landscape of lincRNAs has been explored in serval clades of eukaryotic species, such as the Brassicaceae family [32], monocots [14] and vertebrates [5–7,33,51]. These studies found that, compared to PCGs, lincRNAs are shorter in length, have fewer exons, show lower expression levels and higher tissue specificity. In addition, primary sequences of lincRNAs diverge faster than that of PCGs in both plants and animals. Therefore, the evolutionary distance between species can significantly influence the number of identified homologous lincRNAs and the length of alignable sequence segments between homologous lincRNAs as demonstrated in this study (**Figure S5**).

LincRNAs might be intermediate molecules between neutrally evolved sequences and protein-coding genes, and their functionality may be conferred by their small conserved motifs. The conserved sequence patches within lincRNAs are potentially important for the functionality of lincRNAs. They provide binding sites for transcription factors and RNA binding proteins (RBPs) and can also be translated into small open reading frames (sORFs) [52]. In this study, we found that binding sites of homoeotic proteins, such as AP1 and SEP3, were enriched in the promoters of the conserved plant lincRNAs with a potential role in flower development. LincRNAs usually show tissue-specific expression patterns; it is thus necessary to use same tissues for expression comparison. Rapid divergence of lincRNA sequences would alter or abolish the functionality of lincRNAs, but study in zebrafish showed that homologous lincRNAs with poor sequence conservation could still retain their conserved functionality [53], suggesting that the functionality of lincRNAs may largely depend on short sequence motifs [54] or their secondary structures. For example, the photomorphogenesis-related lincRNA *HID1* exhibits conserved sophisticated secondary structures between *Arabidopsis* and rice [4]. However, there is no robust evidence for the widespread conservation of lncRNA secondary structures [55,56].

### Transposable elements play important roles in the origin of plant lincRNAs

Gain and loss of lincRNAs in the evolution history of plants are faster than that of PCGs [54]. In this study, we used diverse plant species and RNA-seq datasets in identification of lincRNAs. Differences in the quality of genomes and RNA-seq datasets made it difficult to estimate and compare the exact number of lincRNAs in each plant genome; however, it seems that the number of lincRNAs is positively correlated with the size of genomes, particularly in the plant genomes with high proportion of TEs. We hypothesize that this may be partially explained by diverse contributions of TEs in the origin of lincRNAs. TEs might contribute to the exonization of a portion of lincRNAs just like cases in mRNAs [57], providing transcription start sites, splice sites and polyA sites (Kapusta et al. 2013); subsequently, these TE-derived elements or motifs became the sources of functional elements of lincRNAs [58,59]. We found that a significant number of lincRNAs are associated with TEs in most plant species investigated in this study (Fig. 9A). Some of these TE-associated lincRNAs could actually be direct transcription products of TEs. In most plant families (except Brassicaceae), the top type of TEs associated with lincRNAs was retrotransposons (Fig. 9B), consistent with the previous finding that ancestral TEs play an important role in the origin of lincRNAs [60]. In plants, TE-associated lincRNAs could be induced by abiotic stresses, such as salt and cold treatments [61]. In humans, TEs drive tissue-specific expression in stem cells and thus shape the function and evolution of lincRNAs [62]. Furthermore, sequence similarity between some lincRNAs in different species often overlaps with conserved enhancer elements driving the expression of target genes [63].

### Comparative genomic approaches are ways to understand lincRNAs

Comparative genomic approaches are powerful tools to decipher the functions of genes and molecular mechanisms (mode of action) as demonstrated in functional studies of PCGs and miRNAs. For example, some miRNAs, such as miR156 and miR159, are highly conserved in plants, including non-flowering plants. Comparative genomic analyses have facilitated the identification and functional characterization of the conserved miRNAs in different plant species [64]. This principle should also be applicable for lincRNAs. Identification and functional characterization of lincRNAs in model species such as *A. thaliana* would give opportunities to understand their homologous lincRNAs in non-model organisms that do not have well-defined molecular and genetic tools.

Several approaches have been used to identify homologous lincRNAs in plants. One is whole genome alignment. This has been widely used in the animal field because it is available in the public databases such as UCSC genome browse. However, many potential homologous lincRNAs could be missed out when using this approach as lincRNA homology quite often can only be found in short sequence patches; it is therefore critical to find a suitable cut-off value when applying this approach otherwise the power of this method would be compromised. We compared our lincRNA results with those identified using RNAcode (p > 0.05) and found limited overlapping lncRNAs between the two approaches. The sparse properties of phylogenetic tree of the 26 plant genomes used in this study in many clades and widespread whole genome duplication (WGD) create challenges for the whole genome alignment (WGA) of the 26 plant genomes. That might be the reason why we cannot identify candidate homologous sequences based on comparison of genomes, just like RNAcode, to discriminate coding (P < 0.05) and non-coding (P > 0.05). Another approach is to directly compare sequences using alignment tools, such as blast. This approach is computationally more efficient than the approach of whole genome alignment. In addition, based on the syntenic relationship of neighbouring PCGs, positional conservation can also be used to identify homologous lincRNAs. However, if the intergenic region of interest contains multiple lincRNAs, additional information would be required to determine the authentic homologous lincRNAs. Conservation at both sequence and syntenic position would strongly suggest homologous relationship, but the number of such lincRNAs is very small (Fig. 6A**, Figure S5**), presumably due to rapid sequence divergence and/or disruption of syntheny by multiple rounds of whole genome duplication and other forces of genome rearrangement.

While some lincRNAs identified in this study could be false positives, those conserved in multiple species provide resources for identification of their homologs in the newly sequenced plant genomes and candidates for functional characterization. Additionally, many excellent algorithms have been developed for better aligning and comparing lincRNA sequences, which would enhance sequence homology-based identification of lincRNAs. Non-synonymous to synonymous changes (dN/dS) are often used to evaluate evolutionary constraints on PCGs, but its application in lincRNAs is still absent.

## Methods

### 1. Genome-wide identification of lincRNAs

We selected 26 plant species, including six non-flowering plant species, in identification of lincRNAs. The 20 flowering plant species are *Amborella trichopoda* (Atr), *Arabis alpine* (Aal), *Arabidopsis lyrata* (Aly), *Arabidopsis thaliana* (Ath), *Brassica juncea* (Bju), *Brassica napus* (Bna), *Brassica oleracea* (Bol), *Brassica rapa* (Bra), *Capsella rubella* (Cru), *Cicer arietinum* (Car), *Citrullus lanatus* (Cla), *Cucumis sativus* (Csa), *Fragaria vesca* (Fve), *Glycine max* (Gma), *Oryza sativa* (Osa), *Nelumbo nucifera* (Sacred Lotus or Slo), *Solanum lycopersicum* (Sly), *Tarenaya hassleriana* (Tha), *Vitis vinifera* (Vvi) and *Zea mays* (Zma). The six non-flowering plant species are *Azolla filiculoides* (Afi), *Chlamydomonas reinhardtii* (Cre), *Marchantia polymorpha* (Mpo), *Physcomitrella patens* (Ppa), *Selaginella moellendorffii* (Smo) and *Volvox carteri* (Vca). For each plant species, 12 (Aal) to 899 (Zma) RNA-seq datasets generated from different tissues (**Supplemental Table S1**) were collected from the public databases (e.g. NCBI SRA and EBI ENA).

We used a developmental time series of RNA-seq datasets generated from inflorescence, which were harvested at 0, 2, 4 and 8 days after induction with dexamethasone, of *Arabidopsis thaliana* with the AP1-based floral synchronized system [65]. Owing to a subset of lincRNAs lacking polyA tails, total RNA-seq libraries from the same developmental time points described above were also generated using the AP1-GR-based floral induction system to identify lincRNAs (SRA accession number: PRJNA610830).

To identify lincRNAs, RNA-seq reads of each plant species were mapped to its reference genome by hisat2 [66] with default parameters. The mapped reads of each replicate/sample were assembled by stringtie [67] to get assembled transcripts, which were merged together with the stringtie merge module to obtain a set of merged transcripts of each plant species. The merged transcripts of each plant species were compared with the annotated protein-coding genes (PCGs) of the corresponding species to filter out protein-coding genes by the stringtie gffcompare module. The remaining transcripts were further filtered to remove transcripts less than 200-nt, with the predicted longest ORF encoding a peptide longer than 100 amino acids or showing similarity with the protein in the non-redundant protein database (NR) of NCBI based on blastx (E < 10e^−10^). The remaining transcripts were further evaluated for their protein coding potential using the software CPC2 [68] to get the final long non-coding transcripts which were classified into long intergenic non-coding RNAs (lincRNAs), intronic RNAs and antisense lncRNAs according to their genomic position relative to PCGs. Given that most RNA-seq datasets used were generated using non-strand-specific protocols, our following analyses were focused on lincRNAs.

### 2. Estimation of the expression level of lincRNAs

For each plant species, the identified lincRNAs were merged together with its annotated PCGs to obtain the reference transcripts used for indexing with kallisto [69]. Expression levels of both lincRNAs and PCGs were determined by kallisto [69] with the default parameters. For the samples with replicates, the expression levels were estimated based on the average value of the replicated samples. We only retained lincRNAs and PCGs with a TPM > 0.5 as a high percentage of lincRNAs are lowly expressed (<0.5 TPM) (**Figure S11**). However, we are aware that certain noisy transcripts could not be completely removed even using all the above filters [70,71].

### 3. Identification of lincRNA family, homologous lincRNAs and lincRNA evolutionary age group

The repeats masked lincRNA sequences from each plant species were reciprocally compared with each other by BLAST 2.4.0+ (-evalue 1e-5 -num_threads 10 -max_target_seqs 1 -word_size 8 -strand plus -outfmt 6). LincRNA sequences of two plant species with an alignment E-value < 1e-5 were considered to be the best hits and were considered to be homologs [5]. To identify the lincRNA family, an lincRNA sequence similarity network was built to connect homologous lincRNAs from each species. An unsupervised graph cluster algorithm (MCL, https://micans.org/mcl/) was then used to identify the lincRNA cluster within the constructed network (with the parameter: – abc -I 2.0). Each cluster of homologous lincRNAs was designated an lincRNA family that was then assigned to one of the three types of families: one2one, one2many and many2many, based on the number of homologous lincRNAs in the plant species from which the lincRNA(s) were identified. If an lincRNA has only a single homolog in all plant species with the homologous lincRNA identified, the cluster containing these homologous lincRNAs was defined as an one2one family; if an lincRNA has multiple homologs (≥2) in at least one of the plant species, the cluster containing the homologous lincRNAs was defined as an one2many family; if an lincRNA has multiple homologs (≥2) in all plant species with the homologous lincRNAs, the cluster containing the homologous lincRNAs was defined as a many2many family.

The MCScanX [72] software was used to identify syntenic regions between two species based on pairwise comparisons. PCGs within the syntenic regions were used to define syntenic (conserved) lincRNAs between the two corresponding species. We considered three PGCs at each side of a given lincRNA. An lincRNA that was found in two plant species, flanked by a minimum of one syntenic PCG on each side and had a minimum of three syntenic PCGs was defined as syntenic lincRNA [43].

A set of different criteria were used to identify conserved lincRNAs of different evolutionary age groups across different levels of plant lineages, including Plants, Angiosperms, Monocots, Eudicots and Brassicaceae. LincRNAs conserved in the evolutionary age group of Plants should have a homolog in *Amborella trichopoda*, at least a homolog in one of the non-flowering plants, one of the eudicots and one of the monocots (i.e. Atr& (Ath|Aly|Cru|Bol|Bna|Bra|Bju|Aal|Cla|Csa|Car|Gma|Fve|Vvi|Sly|Slo) & (Osa|Zma) & (Ppa|Mpo|Cre|Vca|Smo|Afi)). LincRNAs conserved in the evolutionary age group of Angiosperms should have a homolog in *Amborella trichopoda*, at least one homolog in eudicots and one homolog in monocots (i.e. Atr& (Ath|Aly|Cru|Bol|Bna|Bra|Bju|Aal|Cla|Csa|Car|Gma|Fve|Vvi|Sly|Slo) & (Osa|Zma)). LincRNAs conserved in the evolutionary age group of Moncots_Eudicots (i.e. both monocots and eudicots) should have at least one homolog in both monocots and eudicots (i.e. (Ath|Aly|Cru|Bol|Bna|Bra|Bju|Aal|Cla|Csa|Car|Gma|Fve|Vvi|Sly|Slo) & (Osa|Zma)). LincRNAs conserved in the evolutionary age group of Eudicots should have a homolog in Sacred Lotus (*Nelumbo nucifera*, a basal eudicot), at least one homolog in other eudicots (i.e. Slo & (Ath|Aly|Cru|Bol|Bna|Bra|Bju|Aal|Cla|Csa|Car|Gma|Fve|Vvi|Sly)). LincRNAs conserved in the evolutionary age group of Monocots should have a homolog in both *O. sativa* and *Z. mays* (Osa|Zma).

LincRNAs conserved in the evolutionary age group of Brassicaceae should have a homolog in at least two species of Brassicaceae and also have at least one homolog in Brassicaceae lineage I (Ath, Aly, and Cru) and II (Bol, Bra, Bna, Bju, and Aal), respectively.

Old lincRNAs were defined as those found in the evolutionary age groups of Plants, Angiosperms and Eudicots, while young lincRNAs were defined as those found in the evolutionary age group of Brassicaceae.

### 4. Identification of peaks of histone modification and TF binding sites overlapping with lincRNA and PCGs

Peak files of histone modifications and TF-binding sites were obtained from the ChIP-Hub database [73]. The peaks overlapping with the 1-kb upstream/downstream regions of lincRNAs and PCGs were retrieved by the intersect function of the bedtools v2.25.0. The frequency of lncRNAs or PCGs with histone modification or TF-binding sites was calculated by the number of overlapping sites/the total number of lincRNAs or PCGs.

### 5. Construction of co-expression network involving lincRNAs and PCGs

The co-expression network of lincRNAs and PCGs was constructed individually for *A. thaliana, A. lyrata, Capsella rubella, Brassica napus, Marchantia polymorpha, O. sativa* and *Z. mays* using WGCNA [74]. First, PCGs and lincRNAs with a low coefficient of variation (CV <0.7) among samples were filtered out [75]. The expression level (TPM) of lincRNAs and PCGs was then log2 transformed and normalized into z-score. The soft power of nine was used to fit the scale-free topology of the co-expression network. The default parameters of the dynamic tree were used to get modules of the co-expression networks. The eigengenes of the modules were computed from the first component of the module expression matrix. The PCGs within modules were set as the input of GO enrichment analysis by GOseq [76]. Visualization of the co-expression network was done by Cytoscape [77].

### 6. Identification of transposable elements (TEs) overlapping with lincRNAs

TEs from *A. thaliana* were downloaded from TAIR10 (https://www.arabidopsis.org/download_files/Genes/TAIR10_genome_release/TAIR10_transposable_elements/TAIR10_Transposable_Elements.txt). TEs in other plant genomes were identified by EDTA [78](https://github.com/oushujun/EDTA). The parameters used in TE identification for plant species other than rice and maize were as follows: EDTA.pl – genome genome.fasta – species others – cds cds.fa – anno 1 – threads 20. For rice and maize, the ‘–species’ parameter was set Rice and Maize, respectively. The function of intersect of the bedtools v2.25.0 was used to identify lincRNAs and PCGs intersecting with TEs using the criterion of ≥1-nt overlapping.

## Supplementary Material

Supplemental Material

## Data access

RNA-seq libraries from *A. thaliana* have been deposited in NCBI SRA under the accession number of PRJNA610830. All other datasets were downloaded from NCBI and are presented in Supplemental Table S1 (https://data.mendeley.com/v1/datasets/jhk27dbwrp/draft?a=cf6c6bba-2ebc-45d9-b264-f59f70bc5207).
